# Correlation between the rate of morphological changes and rupture of intracranial aneurysms during one cardiac cycle analyzed by 4D-CTA

**DOI:** 10.3389/fneur.2023.1235312

**Published:** 2023-10-02

**Authors:** Binghao Wang, Chengen Shen, Zhongzhou Su, Xiaohu Nie, Jingjing Zhao, Sheng Qiu, Yuntao Li

**Affiliations:** ^1^Department of Neurosurgery, Huzhou Central Hospital, Affiliated Huzhou Hospital, Zhejiang University School of Medicine, Huzhou, China; ^2^Huzhou Key Laboratory of Basic Research and Clinical Translation for Neuromodulation, Huzhou, China; ^3^Department of Neurosurgery, Fourth Affiliated Hospital of Harbin Medical University, Harbin, China; ^4^Department of Hospital Infection Control, Huzhou Central Hospital, Affiliated Huzhou Hospital, Zhejiang University School of Medicine, Huzhou, China

**Keywords:** 4D-CTA, intracranial aneurysm, morphological change rate, rupture factors, cardiac cycle

## Abstract

**Objective:**

This study aimed to analyze the relationship between the rate of morphological changes and intracranial aneurysm rupture during the cardiac cycle.

**Methods:**

Eighty-four patients with intracranial aneurysms were retrospectively analyzed and divided into the rupture (42 cases) and unruptured (42 cases) groups. Four-dimensional computed tomography angiography (4D-CTA) was performed to collect quantitative parameters of aneurysm morphology and calculate the morphological change rate. The potential factors associated with aneurysm rupture were determined by comparing the general clinical data and rate of change in the location and morphology of the aneurysm between the two groups.

**Results:**

Each morphological change rate in the rupture group was generally higher than that of the unruptured group. The rate of dome height change and aneurysm volume change were independent factors associated with aneurysm rupture. ROC curve analysis revealed that the diagnostic accuracy of the aneurysm volume change rate was higher. When the volume change rate was 12.33%, the sensitivity and specificity of rupture were 90.5 and 55.8%, respectively.

**Conclusion:**

The rate of change in dome height and volume of intracranial aneurysms during one cardiac cycle were independent factors associated with aneurysm rupture.

## Introduction

Intracranial aneurysm is a relatively common cerebrovascular disease resulting from pathological weakness of the intracranial artery wall, leading to gradual abnormal bulging under the impact of blood flow and other factors. Non-traumatic subarachnoid hemorrhage (SAH) is mainly caused by the rupture of intracranial aneurysms and accounts for 75%−85% of cases (1). Owing to the hazardous nature of intracranial aneurysms, they frequently have grave consequences. The fatality rate for initial hemorrhage is as high as 35%, and even after survival, most patients still suffer from severe disability, resulting in significant clinical and socioeconomic burdens (2). With the continuous advancement and widespread application of non-invasive imaging technology, the detection rate of intracranial aneurysms has been steadily increasing annually. The prevalence of unruptured intracranial aneurysms (UIA) is as high as 7% in the population; however, the actual risk of rupture is <1% (3, 4). Prophylactic treatment for these UIAs may lead to surgery-related complications such as intraoperative rupture and infarction. On the other hand, conservative treatment carries the risk of late rupture. Determining the higher risk between treatment and rupture is a major challenge for clinicians. Therefore, precise and timely differentiation between unstable and stable UIAs as well as accurate intervention are crucial components in the field of cerebrovascular disease diagnosis and treatment.

Recently, numerous risk factors for intracranial aneurysms with a high risk of rupture have been reported. These include general clinical characteristics of patients, such as gender, age, smoking, and hypertension (5), as well as morphological features of intracranial aneurysms, such as maximum diameter and aspect ratio (AR) (6, 7). Observational studies on unruptured intracranial aneurysms have demonstrated that the morphological characteristics associated with aneurysm rupture undergo significant changes during aneurysm growth, resulting in a marked increase in annual rupture rates (8, 9). The blood flow between the aneurysm and the parent artery is continuous, resulting in changes in morphology with each pulsation of the parent artery. We postulate that aneurysm pulsation during the cardiac cycle induces morphological changes, which is also associated with aneurysm rupture. With recent advancements in imaging technology, it has become feasible to monitor the dynamic changes in aneurysms caused by pulse pressure from the beating artery of the heart. Current dynamic studies primarily focus on the significance of irregular pulses in aneurysms and predicting the risk of rupture during clipping. However, there is a dearth of research examining dynamic morphological changes in aneurysms for risk assessment purposes (10–12). Four-dimensional computed tomography angiography (4D-CTA) was used in this study to examine the correlation between morphological changes in intracranial aneurysms during the cardiac cycle and aneurysm rupture, aiming to explore potential factors associated with intracranial aneurysms rupture.

## Objects and methods

### Objects

This study involved a retrospective analysis of 84 patients with intracranial aneurysms treated with minimally invasive neurosurgery at the Fourth Affiliated Hospital of Harbin Medical University from October 2015 to October 2020. All patients were Han from Northeast China who provided written informed consent. This study was approved by the hospital ethics review committee.

The inclusion criteria were as follows: (1) patients with 4D-CTA scans, (2) definite diagnosis of intracranial aneurysm, (3) clear image without artifacts, and (4) saccular aneurysm. The exclusion criteria were as follows: (1) partial or complete vascular interventional embolization or craniotomy aneurysm clipping treatment, (2) aneurysms with other cerebrovascular malformations, (3) traumatic and infectious aneurysms, dissecting aneurysms, and fusiform aneurysms, and (4) blurred image or missing data.

The patients were divided into two groups based on their clinical presentation and initial CT scan findings: (1) rupture group, involving patients with clinical symptoms caused by rupture and bleeding and also intracranial hemorrhage or subarachnoid hemorrhage (SAH) confirmed by head CT or lumbar puncture and (2) unruptured group, involving patients with clinical symptoms due to reasons other than rupture or aneurysms accidentally found during examination of other intracranial diseases.

### Acquisition of morphological, clinical, and positional features

**Figure 1:**
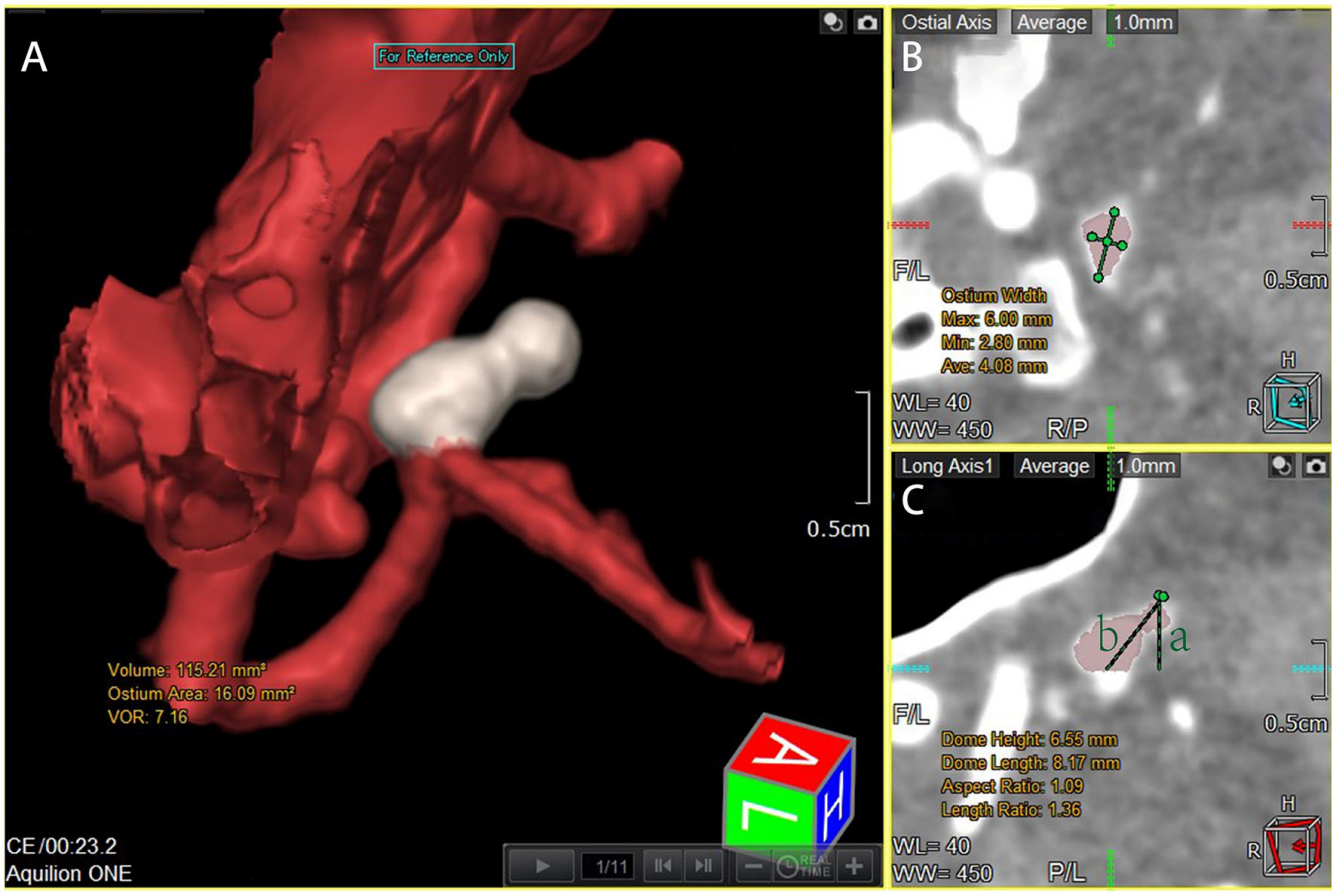
Measurement of dome height, length, ostium width, ostium area, and aneurysm volume. All images were rotationally reconstructed at multiple levels, with the entire aneurysm displayed at the same location to reduce measurement error. **(A)** Automated tracing of the boundaries of the aneurysm after multislice rotational reconstruction demonstrates the ostium area and aneurysm volume. **(B)** Measurement of the ostium width under tomography. **(C)** Another layer perpendicular to B measuring the dome length and height, and line a represents dome height and line b represents dome length.

**Figure 2:**
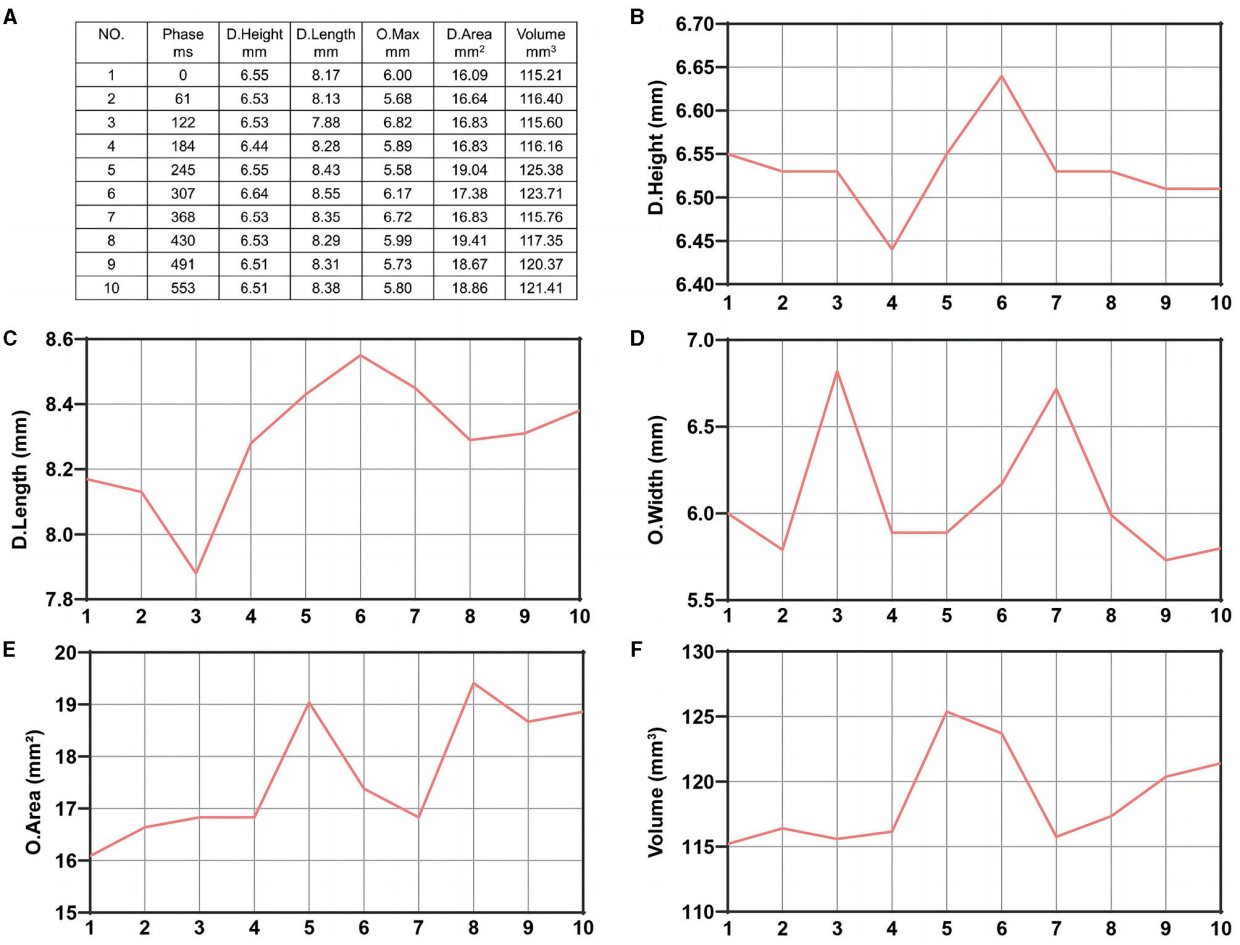
Ten data sets of morphological change curves during one cardiac cycle of the same aneurysm plotted by the software. **(A)** Ten data sets of morphological changes from one aneurysm during one cardiac cycle. **(B)** Dome height change. **(C)** Dome length change. **(D)** Ostium width change. **(E)** Ostium area change. **(F)** Aneurysm volume change.

The patients were scanned using a CT Aquilion One Vision (Toshiba Medical Co., Japan) with the following parameters: 100 kV tube voltage, 105 mA tube current, 0.275 s/turn, slice thickness of 0.5 mm, 16 cm range, and one heartbeat. The scanning mode simulated coronary CTA scanning, and the scanner was connected to an electrocardiogram monitor. At the time of scanning, 55 mL of contrast medium (three generations manifest 350 mg I/ml; Guerbet, France) was injected via the cubital vein using a double-barrelled syringe at a rate of 4.5 ml/s. Ten CT volume data sets were acquired at 10% of the R–R interval within one cardiac cycle. The rotational image of intracranial arteries was reconstructed using the 4D cerebral artery morphological analysis workstation, and the boundaries of the aneurysms were automatically traced by the software to measure the height, length, ostium width, ostium area, and aneurysm volume (Figure 1). The corresponding 10 sets of morphological data for each aneurysm were recorded, and the change curves were plotted (Figure 2). The rate of change of individual morphological features was calculated as (maximum value of 10 phases – minimum value of 10 phases)/mean value of 10 phases (11). We used the maximum diameter to represent aneurysm size, which was measured as the maximum distance from the tip of the aneurysm to the midpoint of its neck, following aneurysm length measurements. The aspect ratio (AR) is the ratio of the height of an aneurysm to the width of its neck, which is equal to the height/ostium width.

The patient's clinical characteristics, including age, sex, hypertension, coronary heart disease (CHD), and type II diabetes, were obtained from inpatient medical records. The location of intracranial aneurysms was also obtained from the patient's imaging reports.

### Statistical analysis

Data were analyzed using SPSS26.0 software. Metrology data were expressed as mean ± standard deviation (SD) using the Shapiro–Wilk analysis after conforming to a normal distribution. The independent samples *t*-test was used to compare two groups, non-conforming normal distribution was expressed as median, and the Wilcoxon rank sum test was used to compare two groups. The count data were expressed as the number of cases and constituent ratio, and the χ^2^ test was used to compare the two groups. Variables that were significant between the two groups were selected for binary logistic regression analysis based on univariate analysis, and α = 0.05 was set as the testing criterion. Finally, the independent factors derived from the regression analysis were subjected to ROC curve analysis to calculate their sensitivity and specificity for diagnosing ruptured aneurysms and to obtain the best factors and judgment threshold.

## Results

### Univariate comparison of the clinical location and morphological features of the groups

A total of 42 intracranial aneurysms from 42 patients in the rupture group and 43 intracranial aneurysms from 42 patients in the unruptured group (one patient had a mirror image aneurysm located in the left and right posterior communicating arteries) were analyzed. The analysis revealed no significant differences between the groups in terms of patient clinical data, aneurysm location distribution, and morphological features (*P* > 0.05, Tables 1–3). However, the rates of each morphological change were significantly different between the two groups, and the rates of morphological changes in intracranial aneurysms during the cardiac cycle were generally higher in the ruptured group than in the unruptured group (*P* < 0.05, Table 4).

**Table 1:** Comparison of clinical characteristics among patients with intracranial aneurysms.

**Characteristic**	**Ruptured (*n* = 42)**	**Unruptured (*n* = 42)**	***P*-value**
**Sex**			
Male	16 (38.1%)	13 (31.0%)	0.491
Female	26 (61.9%)	29 (69.0%)	
Age, mean ± SD	58.17 ± 10.513	60.76 ± 8.781	0.223
Hypertension, *n* (%)	18 (42.9%)	16 (38.1%)	0.657
CHD, *n* (%)	6 (14.3%)	4 (9.5%)	0.500
Type II diabetes, *n* (%)	4 (9.5%)	5 (11.9%)	0.724

**Table 2:** Location distribution of intracranial aneurysms.

**Location**	**Ruptured (*n* = 42)**	**Unruptured (*n* = 42)**	***P*-value**
Middle cerebral artery (MCa)	7 (16.7%)	14 (32.6%)	0.236
Anterior communicating artery (ACoA)	10 (23.8%)	6 (14%)	
Posterior communicating artery (PCoA)	22 (52.4%)	16 (37.2%)	
Anterior choroidal artery (AChA)	1 (2.4%)	1 (2.3%)	
Ophthalmic artery (OA)	1 (2.4%)	4 (9.3%)	
Vertebrobasilar arteries (VBA)	1 (2.4%)	2 (4.7%)	

**Table 3:** Comparative analysis of morphological characteristics of intracranial aneurysms.

**Morphology**	**Ruptured (*n* = 42)**	**Unruptured (*n* = 43)**	***P*-value**
Aneurysm size, mean ± SD, mm	5.413 ± 3.589	5.796 ± 3.375	0.613
Aneurysm neck, mean ± SD, mm	6.059 ± 2.572	5.627 ± 2.340	0.420
Aneurysm height, mean ± SD, mm	5.859 ± 3.292	7.366 ± 7.922	0.255
AR, mean ± SD, mm	1.085 ± 0.625	1.541 ± 2.012	0.163

AR, aspect ratio.

**Table 4:** Wilcoxon rank sum test for the rate of each morphological change.

**Group**	**Dome height change rate (%)**	**Dome length change rate (%)**	**Ostium width change rate (%)**	**Ostium area change rate (%)**	**Aneurysms volume change rate (%)**
Ruptured	8.342	20.502	14.065	19.272	24.620
Unruptured	5.675	9.156	7.119	9.082	11.558
*P*-value	0.042	0.001	0.000	0.002	0.000

### Binary logistic regression analysis

**Figure 3:**
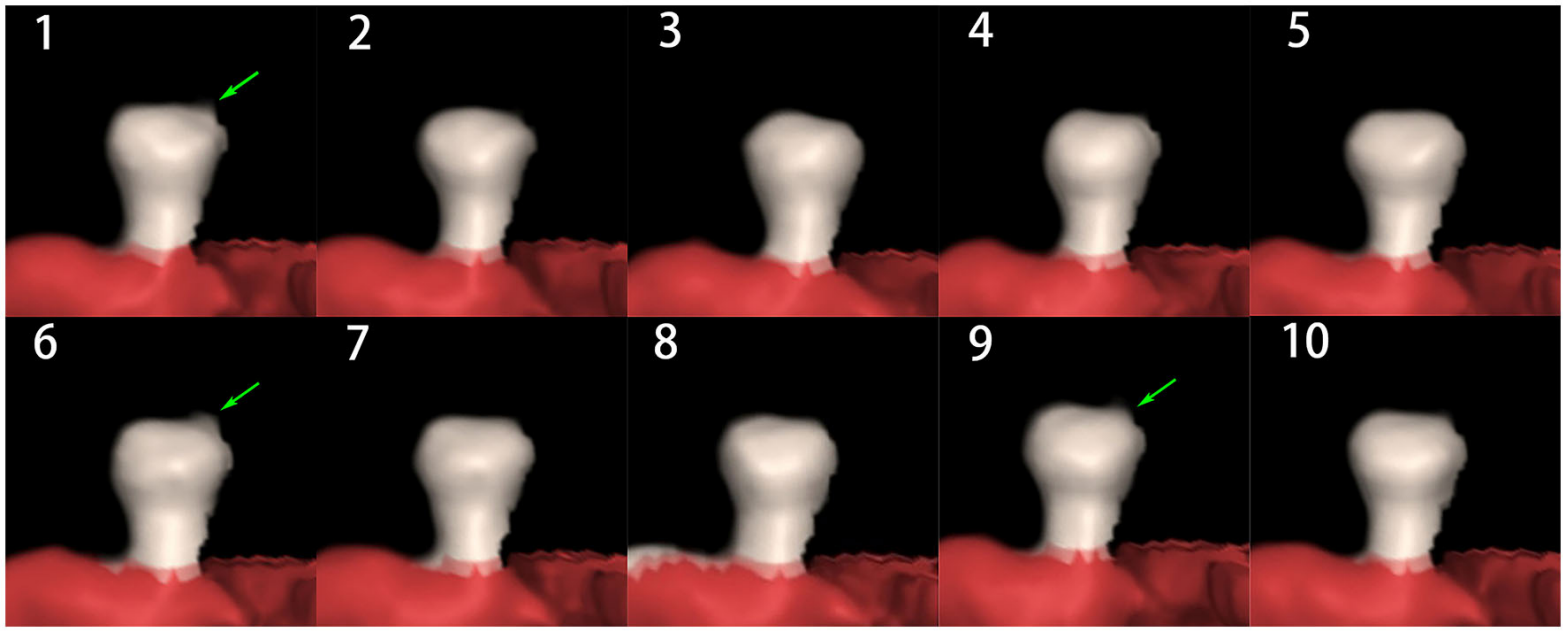
4D-CTA images in one cardiac cycle showing increased volume change (green arrow) in a 65-year-old woman with a ruptured aneurysm.

Multivariate binary logistic regression analysis of the indicators that were statistically significant on univariate analysis demonstrated that a high dome height change rate and aneurysm volume change rate were independent factors associated with intracranial aneurysm rupture (*P* < 0.05) and that a high aneurysm volume change ratio was positively associated with intracranial aneurysm rupture (Table 5, Figure 3).

**Table 5:** Multivariate binary logistic regression analysis of factors influencing intracranial aneurysms rupture.

**Variable**	***B***	**OR (95% CI)**	***P*-value**
Dome height change rate	−0.058	0.944 (0.891–1.000)	0.049
Dome length change rate	0.032	1.033 (0.992–1.075)	0.116
Ostium width change rate	0.08	1.008 (0.985–1.032)	0.497
Ostium area change rate	−0.001	0.999 (0.992–1.006)	0.739
Aneurysms volume change rate	0.071	1.073 (1.022–1.127)	0.005

OR, Odds ratio; B, partial correlation coefficients.

### ROC curve analysis

**Figure 4:**
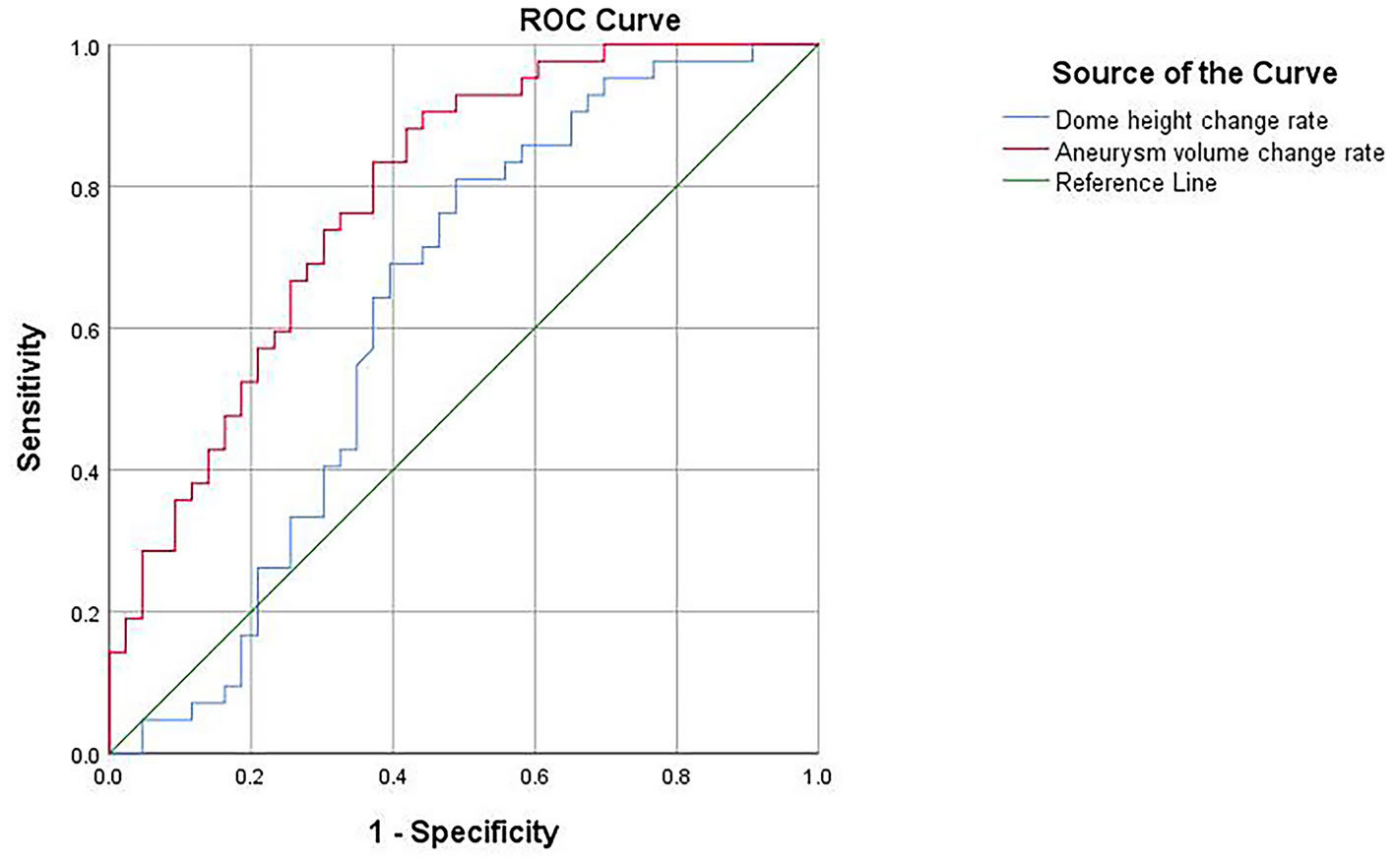
ROC curve for the diagnosis of ruptured aneurysms.

A ROC curve was plotted for the results of the binary logistic regression analysis, yielding an area under the curve of 0.628 for the dome height change rate, with a cutoff value of 5.68% for the diagnosis of ruptured aneurysms, 81% sensitivity, and 51.2% specificity. The area under the curve of the aneurysm volume change rate was 0.786, with a diagnostic cutoff value of 12.33%, 90.5% sensitivity, and 55.8% specificity; therefore, the aneurysm volume change rate had better diagnostic accuracy (Figure 4).

## Discussion

This was a retrospective study of 4D-CTA for intracranial aneurysm detection. We found that both the rate of dome height change and the rate of aneurysm volume change were independently associated with rupture, and the aneurysm volume change rate demonstrated a good ability to analyze aneurysm rupture. Our findings suggest that the rate of morphological changes detected using 4D-CTA in aneurysms may be a potential clinical indicator associated with aneurysm rupture.

Morphology provides an important foundation for studying the risk of aneurysm rupture, but most studies on aneurysm morphology are static (13, 14). Given that blood flow within intracranial aneurysms is subject to changes in response to cardiac contraction and relaxation, a dynamic study of their morphological alterations would be more advantageous. Univariate analysis revealed that the rate of morphological changes during the cardiac cycle was associated with aneurysm rupture compared with other factors. Furthermore, the ruptured group exhibited a consistently higher rate of morphological changes than the unruptured group. The results of multivariate and ROC analyses demonstrated that the change rate of aneurysm height and volume were independent factors associated with the ruptured status, with a certain ability to analyze the factors contributing to aneurysm rupture, especially for the change rate of volume, which showed superior sensitivity.

The rates of dome height and aneurysm volume changes reflect the degree of morphological changes in a cardiac cycle, which can alter intraluminal hemodynamics, an important factor in the occurrence, growth, and rupture of intracranial aneurysms (15–17).

This study found a negative correlation between the rate of change in dome height and aneurysm rupture, suggesting that a higher rate of change in dome height was associated with a lower aneurysm rupture. Blood entering the aneurysm from the parent artery produces wall shear stress (WSS), which increases with blood flow velocity. The blood flow velocity and WSS were the highest at the ostium of the aneurysm. Slow blood rheology within the aneurysm cavity results in decreased WSS, with the slowest blood flow velocity at the top of the aneurysm corresponding to the lowest WSS values. Both high and low WSS can contribute to aneurysm formation and rupture (18–20). The greater the rate of change in aneurysm height, the more obvious the change in height becomes. The distance from the ostium to the top of the aneurysm increased, resulting in a greater degree of velocity change in blood flow. Although WSS at the top of the aneurysm decreases, there is a fluctuation range owing to the dynamic change in height; therefore, WSS will not be too high or too low. Additionally, the pumping of blood by the heart causes a series of changes in blood flow that leads to fluctuations in the aneurysm height. Generally, WSS variation at the apex of an aneurysm oscillates within a narrow range, creating a corresponding buffer to prevent rupture caused by abrupt changes in WSS at this location.

Kuroda first proposed cardiac cycle-related volume changes in intracranial aneurysms and evaluated the relationship between intracranial aneurysms and the movement of normal intracranial arteries in one cardiac cycle. Although no significant difference existed between unruptured aneurysms and normal intracranial arteries, there was still a slight distinction (21). 4D-CTA is a feasible method for quantitatively analyzing the volume changes of aneurysms during a cardiac cycle, providing a theoretical basis for further research (22, 23). In this study, the comparison between ruptured and unruptured aneurysms revealed a significant difference in volume change rate between the two groups. Furthermore, there is a positive correlation between aneurysm volume change rate and rupture risk, indicating that higher volume change rates increase the likelihood of aneurysm rupture. A study on the pathological changes of intracranial aneurysms found that compared to the normal vascular wall, the aneurysm wall showed the absence of a multilayered vessel wall structure-elastic compartment with an increase in permeability, endothelial cell damage and produces areas of inflammation, and chronic increased inflammation further exacerbates vessel wall defects (24–27). The destruction of elastic fibers is most pronounced at the weak point of the tumor wall, where the expansion rate exceeds that of the normal vessel wall, and rupture is more likely to occur. Some studies suggest that the rupture point of an aneurysm is the same as its pulsatile point on 4D-CTA or the weak point observed during surgery, and an aneurysm with a pulsatile point is more likely to rupture (10, 28, 29). The pulsation point was defined as a protrusion observed in the same area of the aneurysm on at least three consecutive images during the cardiac cycle. The appearance of an abnormal pulsation must be accompanied by an increase in volume change. After a rupture, although small blood clots may temporarily protect by attaching to the site of rupture, the aneurysm wall in this area is always weaker than in other parts and more susceptible to expansion due to the pulsation of blood flow. Therefore, for aneurysms with a high-volume change rate, an early warning indicates the possibility of rupture and necessitates prompt treatment.

Additionally, this study revealed a higher incidence of intracranial aneurysms in women than in men. Some scholars believe that the female gender is not only a risk factor for aneurysm formation but also a risk factor for SAH, which is related to the potential protective participation of sex steroids (6). The aneurysms in the present study occurred at the three most common sites of ruptured aneurysms (MCa, ACoA, and PCoA), possibly because the blood flow changes significantly at the bifurcation of blood vessels, making it easier to form aneurysms (30). Our study accurately reflects the distribution of aneurysms; however, due to the small sample size, no significant difference was observed between the two groups.

This study had several limitations. First, it was a retrospective study conducted at a single center, and most patients included were candidates for preventive therapy, which inevitably led to a selection bias. Second, in the ruptured group, the aneurysm had already ruptured at the time of diagnosis, and 4D-CTA was performed after the rupture. Changes in aneurysm morphology may result from its rupture rather than a causal relationship. These factors are associated with the ruptured status and represent important findings, but they do not necessarily increase the risk of rupture. Consequently, our conclusion may deviate from the actual circumstances. Third, the sample size of the study was limited. The optimal approach would entail a multicenter, large-scale study wherein the same patient is monitored from the initial detection of unruptured aneurysms until rupture, with a comprehensive recording of all morphological data for prospective comparative analysis.

## Conclusion

The rates of change in dome height and aneurysm volume may serve as factors associated with rupture, and the influence of volume change rate is more significant. These influencing factors have the potential to become predictive indicators for intracranial aneurysm rupture.

## Data availability statement

The original contributions presented in the study are included in the article/supplementary material, further inquiries can be directed to the corresponding authors.

## Ethics statement

The studies involving humans were approved by Ethics Committee of the Fourth Affiliated Hospital of Harbin Medical University. The studies were conducted in accordance with the local legislation and institutional requirements. Written informed consent for participation was not required from the participants or the participants' legal guardians/next of kin in accordance with the national legislation and institutional requirements.

## Author contributions

BW and CS wrote the manuscript contributed equally to the article. ZS and XN collected and analyze data. JZ, SQ, and YL conducted the work. All authors contributed to the article and approved the submitted version.
